# Supplementary material for: A systematic review and meta-analysis for association of Helicobacter pylori colonization and celiac disease
Source: PLoS One. 2021 Mar 3;16(3):e0241156. doi: 10.1371/journal.pone.0241156 (PMC7928511; doi:10.1371/journal.pone.0241156)
Supplement: S1 File — (DOCX) [file pone.0241156.s002.docx]

**The included studies for evaluation any association between *Helicobacter pylori* and Celiac disease**

1. Agin M, Batun I, Ozdemir S, Doran F, Tumgor G. Prevalence of *Helicobacter pylori* in Turkish children with celiac disease and its effect on clinical, histopathological, and laboratory parameters. Arch Med Sci AMS. 2019;15: 1475. doi: [10.5114/aoms.2019.83699](https://dx.doi.org/10.5114%2Faoms.2019.83699)
2. Aydogdu S, Cakir M, Ali Yuksekkaya H, Tumgor G, Baran M, Arikan C, et al. Helicobacter pylori infection in children with celiac disease. Scand J Gastroenterol. 2008;43: 1088–1093. https://doi.org/10.1080/00365520802101846
3. Broide E, Sandbank J, Scapa E, Kimchi NA, Shapiro M, Lerner A. The immunohistochemistry profile of lymphocytic gastritis in celiac disease and Helicobacter pylori infection: interplay between infection and inflammation. Mediators Inflamm. 2007;2007.
4. Bayrak NA, Tutar E, Volkan B, Sahin Akkelle B, Polat E, Kutluk G, et al. Helicobacter pylori infection in children with celiac disease: Multi‐center, cross‐sectional study. Helicobacter. 2020;25: e12691. doi:10.1111/hel.12691. <https://doi.org/10.1111/hel.12691>
5. Bayrak NA, Volkan B. Helicobacter pylori infection in children with concurrent Celiac disease and type 1 Diabetes Mellitus. Dig Dis. 2021. https://doi.org/10.1159/000514276
6. Cam S, Ugras M. Children with Celiac disease have less H. pylori gastritis. Helicobacter. WILEY 111 RIVER ST, HOBOKEN 07030-5774, NJ USA; 2018.
7. Ciacci C, Squillante A, Rendina D, Limauro S, Bencivenga C, Labanca F, et al. Helicobacter pylori infection and peptic disease in coeliac disease. Eur J Gastroenterol Hepatol. 2000;12: 1283–1287.
8. Crabtree JE, O’Mahony S, Wyatt JI, Heatley R V, Vestey JP, Howdle PD, et al. Helicobacter pylori serology in patients with coeliac disease and dermatitis herpetiformis. J Clin Pathol. 1992;45: 597–600. doi:10.1136/jcp.45.7.597
9. Dettori I, Dore MP, Marras L, Nieddu S, Manca A, Massarelli G, et al. Is there anything to the reported association between Helicobacter pylori infection and celiac disease? Gut. 2001;49: A42--A42.
10. Diamanti A, Maino C, Niveloni S, Pedreira S, Vazquez H, Smecuol E, et al. Characterization of gastric mucosal lesions in patients with celiac disease: a prospective controlled study. Am J Gastroenterol. 1999;94: 1313–1319.
11. Diamantti A, Maino C, Vazquez H, Pedreira S, Niveloni S, Smecuol E, et al. Celiac disease (CD), lymphocytic gastritis and Helicobacter pylori (HP) infection. A controlled study. Gastroenterology. 1998;114: A363.
12. Dore MP, Salis R, Loria MF, Villanacci V, Bassotti G, Pes GM. Helicobacter pylori infection and occurrence of celiac disease in subjects HLA‐DQ 2/DQ 8 positive: A prospective study. Helicobacter. 2018;23: e12465.
13. Feeley KM, Heneghan MA, Stevens FM, McCarthy CF. Lymphocytic gastritis and coeliac disease: evidence of a positive association. J Clin Pathol. 1998;51: 207–210.
14. Jozefczuk J, Bancerz B, Walkowiak M, Glapa A, Nowak J, Piescikowska J, et al. Prevalence of Helicobacter pylori infection in pediatric celiac disease. Age [years]. 2015;6: 3.0.
15. Józefczuk J, Mądry E, Nowak J, Walkowiak M, Łochocka K, Banasiewicz T, et al. Conflicting results of non-invasive methods for detection of Helicobacter pylori infection in children with celiac disease-a preliminary study. Acta Biochim Pol. 2016;63: 127–130.
16. Karttunen T, Niemelä S. Lymphocytic gastritis and coeliac disease. J Clin Pathol. 1990;43: 436.
17. Konturek PC, Karczewska E, Dieterich W, Hahn EG, Schuppan D. Increased prevalence of Helicobacter pylori infection in patients with celiac disease. Am J Gastroenterol. 2000;95: 3682.
18. Lasa J, ZUBIAURRE I, DIMA G, PERALTA D, SOIFER L. Helicobacter pylori prevalence in patients with celiac disease: results from a cross-sectional study. Arq Gastroenterol. 2015;52: 139–142.
19. Lebwohl B, Blaser MJ, Ludvigsson JF, Green PHR, Rundle A, Sonnenberg A, et al. Decreased risk of celiac disease in patients with Helicobacter pylori colonization. Am J Epidemiol. 2013;178: 1721–1730.
20. Lucero Y, Oyarzún A, O’Ryan M, Quera R, Espinosa N, Valenzuela R, et al. Helicobacter pylori cagA+ is associated with milder duodenal histological changes in Chilean celiac patients. Front Cell Infect Microbiol. 2017;7: 376.
21. Luzza F, Mancuso M, Imeneo M, Mesuraca L, Contaldo A, Giancotti L, et al. Helicobacter pylori infection in children with celiac disease: prevalence and clinicopathologic features. J Pediatr Gastroenterol Nutr. 1999;28: 143–146.
22. Narang M, Puri AS, Sachdeva S, Singh J, Kumar A, Saran RK. Celiac disease and Helicobacter pylori infection in children: Is there any Association? J Gastroenterol Hepatol. 2017;32: 1178–1182.
23. Nejad MR, Rostami K, Yamaoka Y, Mashayekhi R, Molaei M, Dabiri H, et al. Clinical and histological presentation of Helicobacter pylori and gluten related gastroenteropathy. Arch Iran Med. 2011;14: 115–119. doi:011142/AIM.009
24. Rostami-Nejad M, Villanacci V, Mashayakhi R, Molaei M, Bassotti G, Zojaji H, et al. Celiac disease and Hp infection association in Iran. Rev Esp Enfermedades Dig. 2009;101: 850–854. doi:10.4321/S1130-01082009001200004
25. Simondi D, Ribaldone DG, Bonagura GA, Foi S, Sapone N, Garavagno M, et al. Helicobacter pylori in celiac disease and in duodenal intraepithelial lymphocytosis: active protagonist or innocent bystander? Clin Res Hepatol Gastroenterol. 2015;39: 740–745.
26. Uyanikoglu A, Dursun H, Yenice N. Is There any Association Between Celiac Disease and Helicobacter pylori? Euroasian J hepato-gastroenterology. 2016;6: 103.
